# Levosimendan Relaxes Pulmonary Arteries and Veins in Precision-Cut Lung Slices - The Role of K_ATP_-Channels, cAMP and cGMP

**DOI:** 10.1371/journal.pone.0066195

**Published:** 2013-06-18

**Authors:** Annette D. Rieg, Rolf Rossaint, Eva Verjans, Nina A. Maihöfer, Stefan Uhlig, Christian Martin

**Affiliations:** 1 Institute of Pharmacology and Toxicology, Medical Faculty Aachen, Rhenish Westphalian Technical University, Aachen, Germany; 2 Department of Anesthesiology, Medical Faculty Aachen, Rhenish Westphalian Technical University, Aachen, Germany; 3 Department of Pediatrics, Medical Faculty Aachen, Rhenish Westphalian Technical University, Aachen, Germany; Indiana University, United States of America

## Abstract

**Introduction:**

Levosimendan is approved for left heart failure and is also used in right heart failure to reduce right ventricular afterload. Despite the fact that pulmonary arteries (PAs) and pulmonary veins (PVs) contribute to cardiac load, their responses to levosimendan are largely unknown.

**Materials and Methods:**

Levosimendan-induced vasorelaxation of PAs and PVs was studied in precision-cut lung slices from guinea pigs by videomicroscopy; baseline luminal area was defined as 100%. Intracellular cAMP- and cGMP-levels were measured by ELISA and NO end products were determined by the Griess reaction.

**Results:**

Levosimendan relaxed control PVs (116%) and those pre-constricted with an endothelin_A_-receptor agonist (119%). PAs were only relaxed if pre-constricted (115%). Inhibition of K_ATP_-channels (glibenclamide), adenyl cyclase (SQ 22536) and protein kinase G (KT 5823) largely attenuated the levosimendan-induced relaxation in control PVs, as well as in pre-constricted PAs and PVs. Inhibition of BK_Ca_^2+^-channels (iberiotoxin) and K_v_-channels (4-aminopyridine) only contributed to the relaxant effect of levosimendan in pre-constricted PAs. In both PAs and PVs, levosimendan increased intracellular cAMP- and cGMP-levels, whereas NO end products remained unchanged. Notably, basal NO-levels were higher in PVs. The K_ATP_-channel activator levcromakalim relaxed PAs dependent on cAMP/PKA/PKG and increased cAMP-levels in PAs.

**Discussion:**

Levosimendan initiates complex and divergent signaling pathways in PAs and PVs. Levosimendan relaxes PAs and PVs primarily via K_ATP_-channels and cAMP/cGMP; in PAs, BK_Ca_^2+^- and K_v_-channels are also involved. Our findings with levcromakalim do further suggest that in PAs the activation of K_ATP_-channels leads to the production of cAMP/PKA/PKG. In conclusion, these results suggest that levosimendan might reduce right ventricular afterload by relaxation of PAs as well as pulmonary hydrostatic pressure and pulmonary edema by relaxation of PVs.

## Introduction

The Ca^2+^-sensitizer levosimendan reduces mortality in acute heart failure [1]. Levosimendan is also used to treat right heart failure and secondary pulmonary hypertension (PH) [2], since several studies suggested that it decreases right ventricular afterload and mean pulmonary arterial pressure (mPAP) [3], [4]. However, other studies failed to observe such effects [5], [6] and in two patients with idiopathic PH levosimendan even elevated mPAP [7].

These conflicting data raise the possibility that the effects of levosimendan on right ventricular afterload are explained by improved left ventricular contractility rather than by reduced pulmonary vascular resistance (PVR). Except for one study in feline lung lobes [3], the relaxant properties of levosimendan were studied in systemic vessels only [8]–[11]. There, in extrapulmonary vessels, opening of ATP-activated potassium channels (K_ATP_-channels) [12], [13] was identified as a key mechanism in levosimendan-induced relaxation, but Ca^2+^-desensitizing [8] and opening of large conductance Ca^2+^-activated potassium channels (BK_Ca_^2+^-channels), as well as opening of voltage- gated potassium channels (K_v_-channels) [9], [10] were also implicated. However, systemic and pulmonary vessels show remarkable dissimilarities, as is illustrated by their divergent responses to hypoxia, hypercapnia or acidosis [14] and the regulation of endothelial permeability [15]. Furthermore, although differences between PAs and PVs are highly relevant to left- and right-sided heart failure, it is completely unknown whether levosimendan acts differently in both vascular systems which are known for their remarkably distinct behaviour [16], [17]. Notably, PVs contribute up to 40% to PVR [18] and their relaxation would be a useful intervention to reduce pulmonary edema, left ventricular volume-overload and secondary PH.

To clarify whether, where and how levosimendan relaxes pulmonary vessels, we analysed its effects on PAs and PVs in precision-cut lung slices (PCLS), a novel method in pulmonary vascular pharmacology [16]. We chose guinea pigs (GPs), because several studies have indicated that GPs are the best approximation to human lungs when it comes to pulmonary smooth muscle pharmacology [19], [20]. We addressed various signaling mechanisms, e.g. K_ATP_-, BK_Ca_^2+^- and K_v_-channels as well as cAMP- and NO-dependent pathways and report that levosimendan relaxed PAs and PVs via common (K_ATP_-channels, cAMP/cGMP) and different (BK_Ca_^2+^-and K_v_-channels in PAs) mechanisms.

## Materials and Methods

### Guinea Pigs (GPs)

Female Dunkin Hartley GPs (400±50 g; 6–8 weeks old) were obtained from Charles River (Sulzfeld, Germany). All animal care and experimental procedures were performed according to the rules of the Directive 2010/63/EU of the European Parliament. They were approved by the Landesamt für Natur, Umwelt und Verbraucherschutz Nordrhein-Westfalen (approval-ID: 8.87–51.05.20.10.245).

### PCLS

PCLS (GP; n = 46) were prepared as described before [16], [19]. Briefly, intraperitoneal anaesthesia was performed with 95 mg kg^−1^ pentobarbital (Narcoren; Garbsen, Germany) and verified by missing reflexes. Afterwards, the abdomen was opened and the GP exsanguinated. Thereafter, the trachea was cannulated and the diaphragm opened. The lungs were filled with 28–30 ml of 1.5% low melting point agarose (containing 1 µM isoproterenol) as far as a slight resistance develops. The lobes were removed; tissue cores were prepared and cut into 300 µm thick slices with a Krumdieck tissue slicer (Alabama, Munford, AL, USA). PCLS were incubated at 37°C and the medium was changed several times in order to wash out the agarose. PCLS are known to be at least 72 h viable [19], [21].

### Vessel Preparation and Measurement of NO, cAMP and cGMP

For analysis of cAMP/cGMP-production, PAs and PVs were separated out of tissue cores. In contrast, the slices were cut into tissue, containing either the PA or the PV to determine NO end products. Two such tissue pieces together were incubated over 30 minutes with levosimendan (100 µM); controls remained untreated. Supernatants were collected. NO was measured using a NO-kit based on the Griess reaction and nitrite was detected at 550 nM (GENIOS, Tecan, Switzerland).

To measure intracellular cAMP/cGMP, PAs or PVs from tissue cores were cannulated by a plastic catheter (22 gauges), isolated, flushed with levosimendan (100 µM) or levcromakalim (100 µM) and incubated for 30 minutes. Some PAs were also pre-treated with glibenclamide (10 µM for 1 h). Thereafter, vessels were frozen by liquid nitrogen. PAs and PVs were distinguished by their localization, as explained below. Intracellular cAMP/cGMP was quantified with ELISA-kits following the manufacturer’s protocol. For stabilization, all samples and standards were acetylated. To measure cAMP all samples were diluted 1∶2 with 0.1 M HCL. ELISAs were evaluated at 405 nM (GENIOS, Tecan, Switzerland).

### Vessel Size, Identification of the Vessels and Histology

GPs’ pulmonary vessels derived from a central part of the lung and their internal diameter ranged from 500 to 800 µM. PAs and PVs were identified by their anatomical landmarks. PAs accompany the airways and PVs lie aside. In PCLS, this was confirmed with haematoxylin-eosin staining, where PAs show a wrinkled inner lining and a thick media [16], [17].

### Measurements and Imaging

**Figure 1:**
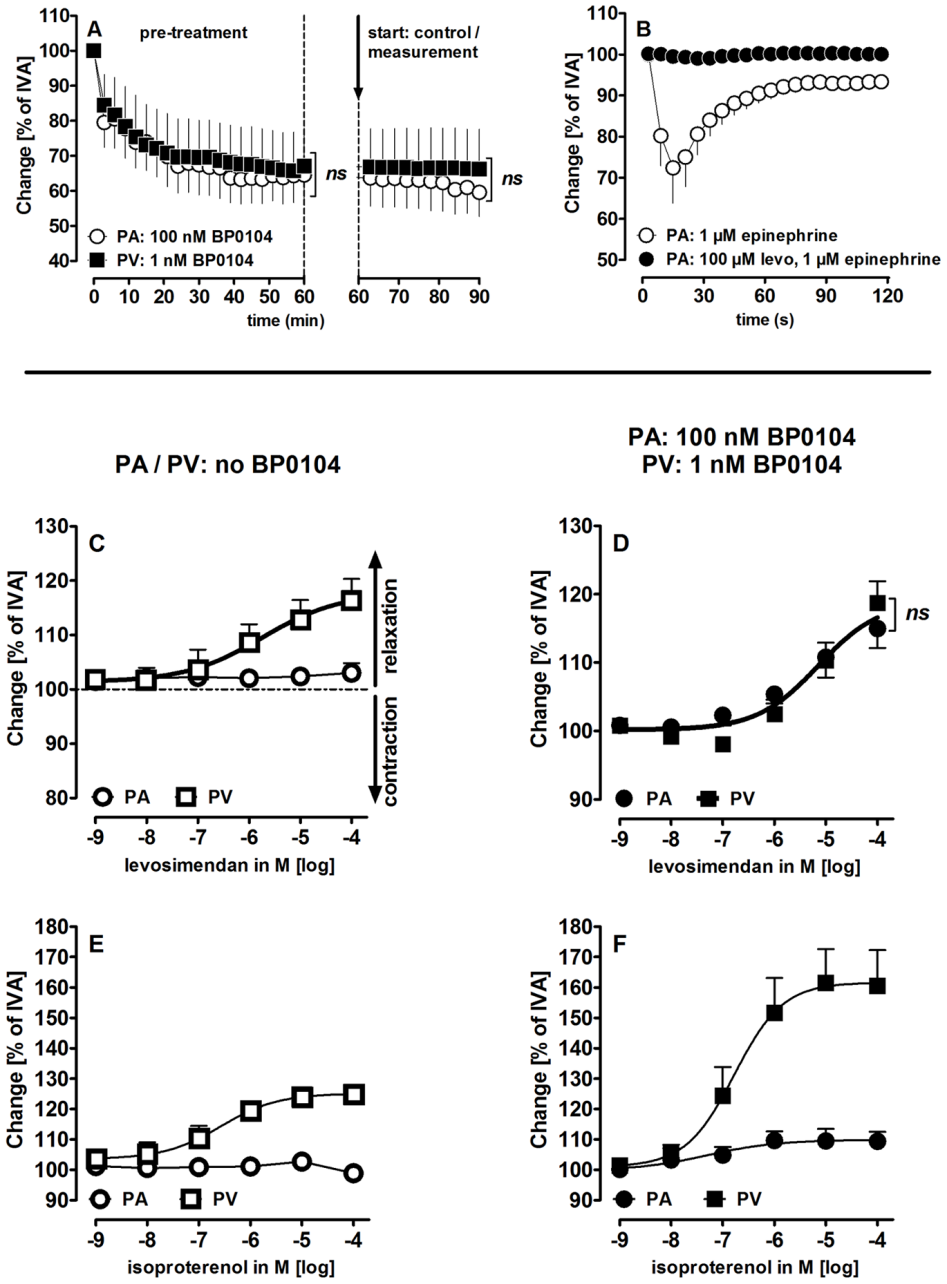
Vasodilating effects of levosimendan (levo) and isoproterenol in pulmonary vessels. **A)** BP0104 in PAs and PVs: (**
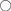
**) PA: 100 nM BP0104 (n = 5); (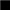) PV: 1 nM BP0104 (n = 5). The dashed line indicates the end of the pre-treatment and the start of the measurements. **B)** Levosimendan prevents epinephrine-induced contraction in PAs: (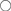) epinephrine 1 µM (n = 5), (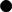) levo 100 µM, epinephrine 1 µM (n = 7); **C)** Levosimendan in PAs/PVs without pre-constriction: (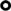) PAs (n = 10); (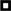) PVs (n = 11); **D)** Levosimendan in pre-constricted PAs/PVs: (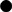) PAs (n = 9); (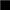) PVs (n = 7); **E)** Isoproterenol in PAs/PVs without pre-constriction: (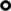) PAs (n = 5) (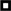) PVs (n = 5) **F)** Isoproterenol in pre-constricted PAs/PVs: (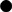) PAs (n = 3); (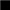) PVs (n = 4). **A)** Statistics was performed by a linear mixed model analysis. **C–D)** The thick solid concentration-response curve share the same EC_50_ value of 5 µM. P<0.05 are considered as significant: *p<0.05, **p<0.01 and ***p<0.001.

The kinetics of all used agents was studied. According to the results, PCLS were exposed 5 minutes to each concentration of the vasodilators (isoproterenol, levosimendan, forskolin or levcromakalim). If pre-constriction was required, they were pre-treated 1 h with the endothelin_A_-(ET_A_)-receptor agonist BP0104. If a signaling pathway was inhibited, PCLS were pre-treated 1 h with the specific inhibitor. If both were required, PCLS were exposed simultaneously to both. Before the measurements, the initial vessel area (IVA) was defined as 100% and any relaxant or contractile effect (BP0104 or inhibitors) was indicated as “Change [% of IVA]”. To compare relaxation of pre-treated vessels, the vessel area was defined after pre-treatment again as 100%. Hence, a vessel area <100% indicates a contractile effect and a vessel area >100% indicates a relaxant effect (Fig. 1C). Concentration-response curves of the vasodilators were performed and the effects were indicated again as “Change [% of IVA]”. In addition, all pre-treatment procedures were indicated in the graphs. We further studied whether PCLS differ in their vascular response dependent on the time-point of drug exposure. We did not find any differences; hence we performed our experiments with PCLS on day one and two after preparation. Prior to the experiments, the reactivity of PAs and PVs was tested in different, but comparable slices by the contractile effect of 1 µM epinephrine (PAs) and by the relaxant effect of 1 µM isoproterenol (PVs). Control experiments were performed on consecutive sections. Pulmonary vessels were imaged and digitised by a digital video camera (Leica Viscam 1280 or Leica DFC 280). The images were analysed with Optimas 6.5 (Media Cybernetics, Bothell, WA).

### Agents and Culture Medium

All agents were bought from Tocris Bioscience (Ellisville, Missouri, USA), except levosimendan and N-nitro-L-arginine methyl ester (L-NAME) which were bought from Sigma-Aldrich (Steinheim, Germany) and BP0104 which was from BIOTRENDS (Wangen, Switzerland). The cAMP/cGMP/NO-kits were purchased from Enzo Life Sciences (Lörrach, Germany). All inhibitors are listed (Table 1).

**Table 1:** Overview of all used inhibitors.

Inhibitor	Target	IC_50_	Used dosage
glibenclamide	K_ATP_-channels	20–200 nM	10 µM
iberiotoxin	BK_Ca_^2+^-channels	10 nM	100 nM
4-aminopyridine	K_v_-channels	0.3–1.1 mM	5 mM
SQ 22536	adenyl cyclase	1.4–200 µM	100 µM
KT 5720	PKA	60 nM	1 µM
L-NAME	NOS	25 µM	100 µM
ODQ	guanylyl cyclase	20 nM	1 µM
KT 5823	PKG	0.23 µM	2 µM

In general, we expect complete inhibition of the target at concentrations about 10 times above the IC_50_ value [26], [44].

### Statistics

**Figure 2:**
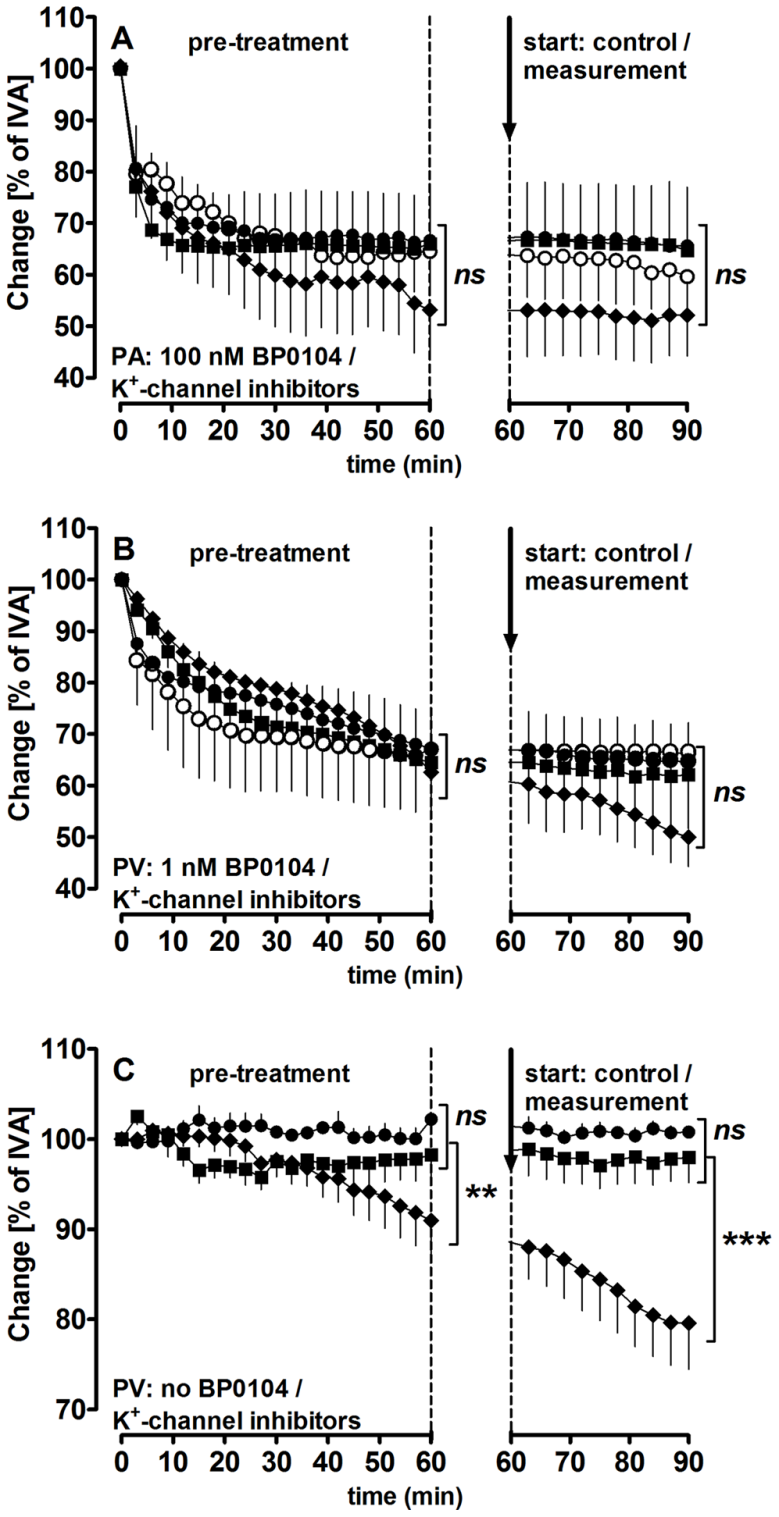
Effect of K+-channel inhibitors on BP0104-induced contraction in PAs/PVs and on control PVs. **A) PAs:** (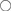) BP0104 (100 nM) (n = 5); (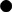) BP0104 (100 nM), iberiotoxin (100 nM) (n = 5); (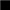) BP0104 (100 nM), glibenclamide (10 µM) (n = 5); (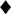) BP0104 (100 nM), 4-aminopyridine (5 mM) (n = 6) **B) PVs:** (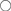) BP0104 (1 nM) (n = 5); (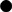) BP0104 (1 nM), iberiotoxin (100 nM) (n = 5); (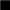) BP0104 (1 nM), glibenclamide (10 µM) (n = 6); (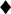) BP0104 (1 nM), 4-aminopyridine (5 mM) (n = 5) **C)** **PVs:** (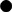) iberiotoxin (100 nM) (n = 3); (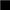) glibenclamide (10 µM) (n = 3); (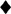) 4-aminopyridine (5 mM) (n = 6). The dashed line indicates the end of pre-treatment. Time course measurements were analysed by a linear mixed model. P<0.05 are considered as significant: *p<0.05, **p<0.01 and ***p<0.001.

Statistics was conducted using SAS software 9.2 (SAS Institute, Cary, North Carolina, USA) and GraphPad Prism 5.01 (GraphPad, La Jolla, USA). The data in Fig. 1A and 2 A, B and C were analysed using a linear mixed model analysis (LMM) with variance components (VC) for the covariance matrix; EC_50_ values were calculated by the standard 4-paramter logistic non-linear regression model. The AIC-criterion was used to select the most parsimonious model, i.e. a common bottom, top, slope and EC_50_ value in the regression model or the covariance matrix with the least number of parameters. Non-parametric analysis was performed by the Mann-Whitney U test. All p-values were adjusted for multiple comparisons by the false discovery rate (FDR) and presented as mean+SEM or - SEM. P<0.05 was considered as significant and (n) indicates the numbers of animals.

## Results

We studied the relaxant effects of the inodilator levosimendan (levo) in control (not pre-constricted) and pre-constricted pulmonary vessels and compared it to the β-receptor agonist isoproterenol.

### Pre-constriction

PAs and PVs were pre-constricted with various concentrations of the ET_A_-receptor agonist BP0104 to identify concentrations that elicit a comparable degree of contraction after 60 minutes. Stable contractions of 62% of IVA were obtained by using 100 nM BP0104 in PAs and 1 nM BP0104 in PVs (Fig. 1A, Table 2).

**Table 2:** Influence of various agonists and inhibitors on the initial vessel area of pulmonary vessel

						p-values			
agents (1 h pre-treatment)	PA mean (%)	n		SEM		vs control		vs BP0104	
BP0104 100 nM	62.1	19		3.1		<0.001			
BP0104 100 nM+glibenclamide 10 µM	68	11		6.1		<0.001		ns	
BP0104 100 nM+iberiotoxin 100 nM	63.7	10		4.6		<0.001		ns	
BP0104 100 nM +4-aminopyridine 5 mM	62.8	10		9.3		<0.001		ns	
glibenclamide	99.4	6		1.6		ns			
iberiotoxin	98.4	7		2.4		ns			
4-aminopyridine (5 mM)	102	5		1.6		ns			
BP0104 100 nM+SQ 22536 (100 µM)	69.9	9		3		<0.001		ns	
BP0104 100 nM+KT 5720 (1 µM)	65.6	8		2.2		<0.001		ns	
L-NAME (100 µM)	97.8	10		0.5		ns			
BP0104 100 nM+L-NAME (100 µM)	47.6	13		4.5		<0.001		<0.05	
BP0104 100 nM+ODQ (1 µM)	48.7	5		7		<0.001		ns	
BP0104 100 nM+KT 5823 (2 µM)	66.2	8		5.3		<0.001		ns	
**agents (1 h pre-treatment)**	**PV mean (%)**		**n**		**SEM**		**p-values**		
							**vs control**		**vs BP0104**
control	99.1	11		0.3					
BP0104 1 nM	62.5	11		5.3		<0.001			
BP0104 1 nM+glibenclamide (10 µM)	64.2	16		5.2		<0.001		ns	
BP0104 1 nM+iberiotoxin (100 nM)	71.7	8		8.3		<0.001		ns	
BP0104 1 nM +4-aminopyridine (5 mM)	67.9	11		5.2		<0.001		ns	
glibenclamide 10 µM	100	9		2.2		ns			
iberiotoxin 100 nM	95.4	10		2.7		ns			
4-aminopyridine 5 mM	89.8	10		2.2		<0.001			
BP0104 1 nM+SQ 22536 (100 µM)	65.1	9		9		<0.01		ns	
BP0104 1 nM+KT 5720 (1 µM)	53.4	10		6.2		<0.001		ns	
SQ 22536 (100 µM)	99.8	8		2.5		ns			
KT 5720 (1 µM)	97.8	11		2.4		ns			
BP0104 1 nM+KT 5720+ KT 5823	64.9	8		6.8				ns	
L-NAME (100 µM)	78.4	26		4.2		<0.001			
ODQ (1 µM)	77.7	5		6.3		<0.001			
KT 5823 (2 µM)	93.8	12		1.5		<0.01			
BP0104 1 nM+L-NAME (100 µM)	59.2	8		7.1		<0.001		ns	
BP0104 1 nM+ODQ (1 µM)	67.6	5		7		<0.001		ns	
BP0104 1 nM+KT 5823 (2 µM)	61.7	9		9.3		<0.01		ns	

The contractile effect of various pre-treatment procedures is indicated after a pre-treatment period of 1 h, prior to the application of levosimendan. Statistics was conducted using the Mann-Whitney U test. All p-values were adjusted for multiple comparisons by the FDR. P<0.05 are considered as significant:

★ p<0.05,

★★ p<0.01 and.

★★★ p<0.001.

### Effects of Levosimendan

Levosimendan relaxed control PVs, but had no effect on control PAs (Fig. 1C). However, it prevented epinephrine-induced contractions in control PAs (Fig. 1B). Furthermore, if pre-constricted with BP0104, levosimendan relaxed both PAs and PVs (Fig. 1D). In all three situations – control veins, pre-constricted PVs and PAs – levosimendan had the same EC_50_-value (5 µM) and Hill slope (0.62). To compare the relaxant potency of levosimendan to a well-known relaxant, we treated PAs and PVs with the β-receptor agonist isoproterenol. Like levosimendan, isoproterenol relaxed naïve PVs (EC_50_∶0.26 µM), but not control PAs (Fig. 1E). Isoproterenol relaxed control and pre-constricted PVs stronger than levosimendan (p<0.001), whereas pre-constricted PAs were relaxed similarly (1F).

### Pulmonary Venous Resting Tone

**Figure 3:**
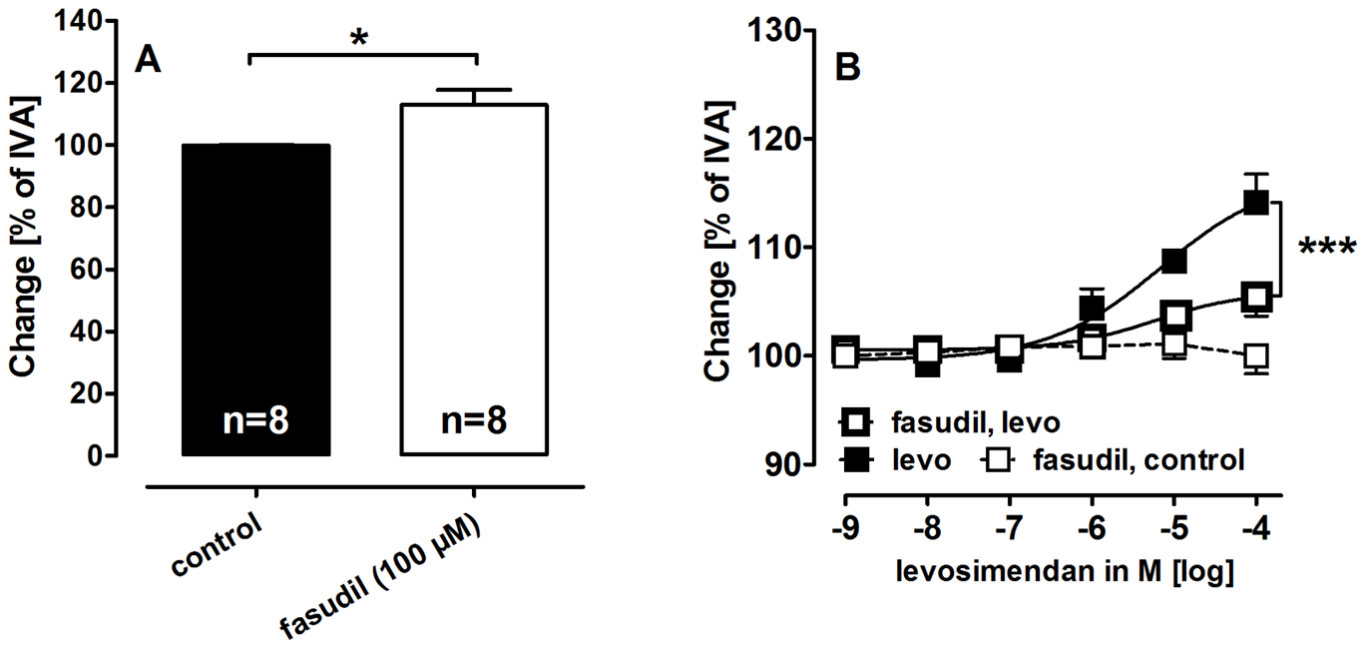
Impact of Rho-Kinase inhibition on the tone of PVs and on the relaxant effect of levosimendan. **A)** Fasudil (100 µM) affects the tone of PVs. **B)** The relaxant potency of levosimendan after pre-treatment with fasudil: (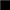) levo (n = 6); (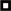) fasudil (100 µM), levo (n = 5); (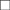) fasudil (100 µM) (n = 4); **A)** Statistics was performed by the Mann-Whitney U test. **B)** Asterics indicate different EC_50_ values. P<0.05 are considered as significant: *p<0.05, **p<0.01 and ***p<0.001.

Obviously, PAs relax to levosimendan and isoproterenol only if pre-constricted; in contrast PVs relax independent from pre-constriction, suggesting that PVs express a certain pulmonary venous resting tone. Inhibition of NO/cGMP/PKG-signaling (Table 2) and K_v_-channels (Table 2, Fig. 2C) increase the pulmonary venous tone, whereas inhibition of cAMP/PKA-signaling (Table 2) does not. To uncover possible mechanisms behind this resting tone, PVs were pre-treated with the Rho-Kinase inhibitor fasudil (100 µM) and thereafter exposed to increasing concentration of levosimendan. Fasudil decreased the tone of PVs and increased the vessel area to 113% of IVA (Fig. 3A). Further, PVs pre-treated with fasudil only relaxed attenuated to levosimendan compared to control PVs (Fig. 3B).

### Role of K^+^-channels in Levosimendan-induced Vasorelaxation

**Figure 4:**
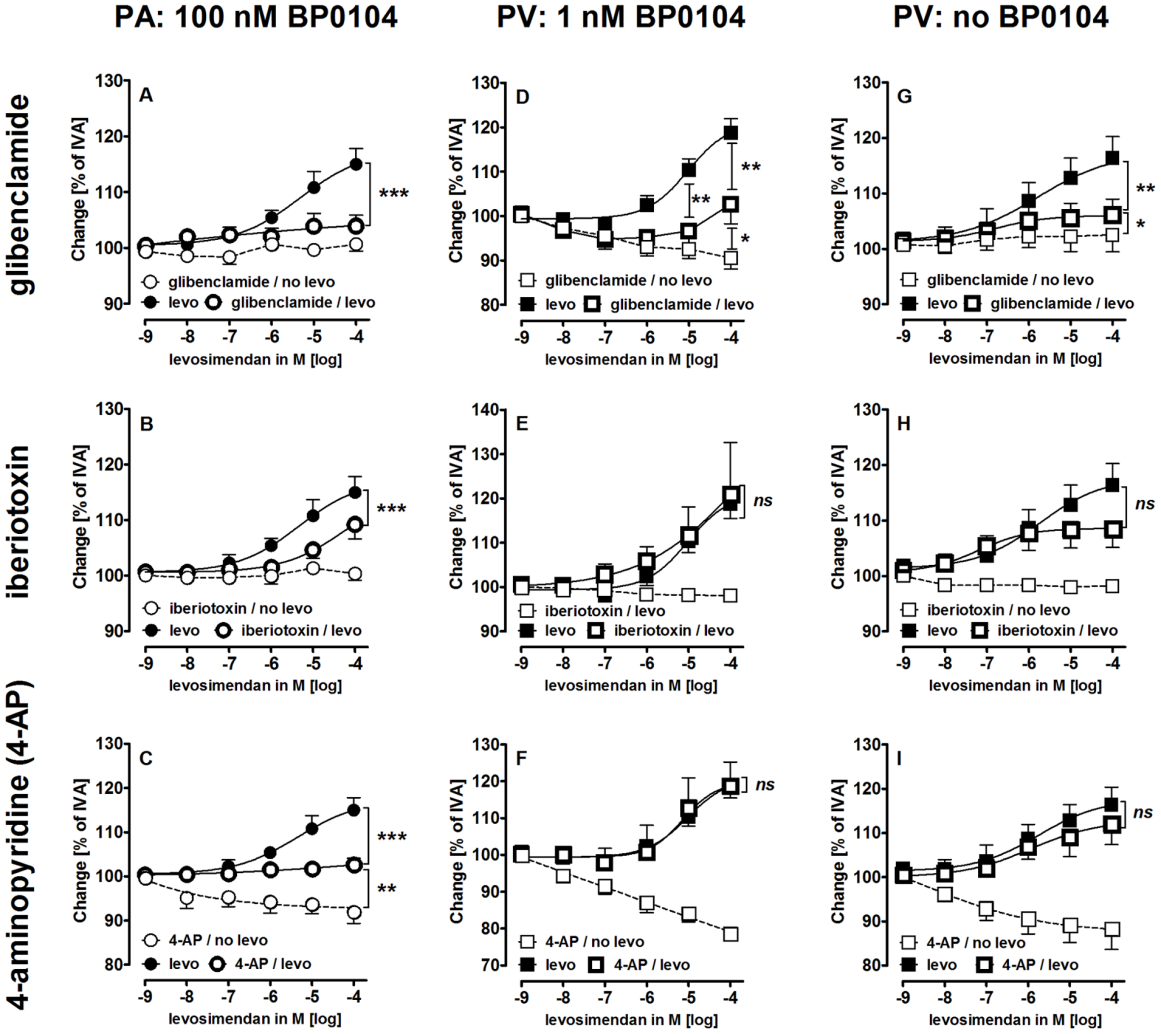
Relaxant effects of levosimendan (levo) in PAs and PVs after K+-channel inhibition. A) PA (100 nM BP0104): (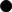) levo (n = 9); (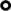) glibenclamide (10 µM), levo (n = 6); (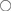) glibenclamide (10 µM) (n = 3); B) PA (100 nM BP0104): (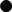) levo (n = 9); (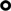) iberiotoxin (100 nM), levo (n = 10); (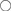) iberiotoxin (100 nM) (n = 3); C) PA (100 nM BP0104): (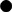) levo (n = 9); (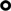) 4-AP (5 mM), levo (n = 6); (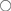) 4-AP (5 mM) (n = 6); D) PV (1 nM BP0104): (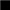) levo (n = 7); (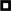) glibenclamide (10 µM), levo (n = 5); (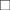) glibenclamide (10 µM) (n = 5); E) PV (1 nM BP0104): (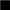) levo (n = 7); (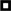) iberiotoxin (100 nM), levo (n = 4); (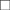) iberiotoxin (100 nM) (n = 3); F) PV (1 nM BP0104): (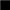) levo (n = 7); (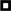) 4-AP (5 mM), levo (n = 4); (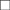) 4-AP (5 mM) (n = 4); G) PV: (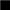) levo (n = 11); (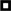) glibenclamide (10 µM), levo (n = 6); (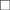) glibenclamide (10 µM) (n = 4); H) PV: (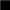) levo (n = 11); (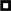) iberiotoxin (100 nM), levo (n = 9); (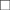) iberiotoxin (100 nM) (n = 3); I) PVs: (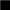) levo (n = 11); (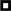) 4-AP (5 mM), levo (n = 5); (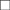) 4-AP (5 mM) (n = 4). A-B/E-H) Asterics indicate different EC_50_ values. C–D) Each corresponding concentration of (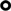) and (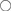) was compared by the Mann-Whitney U test. P<0.05 are considered as significant: *p<0.05, **p<0.01 and ***p<0.001.

To study the role of K^+^-channels in the levosimendan-induced relaxation, the K_ATP_-channel inhibitor glibenclamide (10 µM), the BK_Ca_^2+^-channel inhibitor iberiotoxin (100 nM) and the K_v_-channel inhibitor 4-aminopyridine (5 mM) were used. **Pre-constricted** **PAs and PVs**: In the absence of levosimendan, none of these inhibitors altered the BP0104-induced contraction (Fig. 2A/B; Table 2). **Not pre-constricted PVs**: In the absence of levosimendan, glibenclamide and iberiotoxin did not affect the vascular tone, whereas 4-aminopyridine time-dependently contracted PVs up to 70% after 4 h, but not PAs (Fig. 2C; Table 2). In **pre-constricted** **PAs**, the levosimendan-induced relaxation was reduced by glibenclamide and by iberiotoxin (Fig. 4A/B). Further, in **pre-constricted** **PAs**, 4-aminopyridine (4-AP) showed a small but steady contraction that was partially inhibited by levosimendan (Fig. 4C). In **pre-constricted and non-pre-constricted PVs**, the levosimendan-induced relaxation was reduced by glibenclamide (Fig. 4D/G) but neither by iberiotoxin (Fig. 4E/H) nor by 4-aminopyridine (Fig. 4F/I).

### Role of cAMP/cGMP and NO in Levosimendan-induced Relaxation

**Figure 5:**
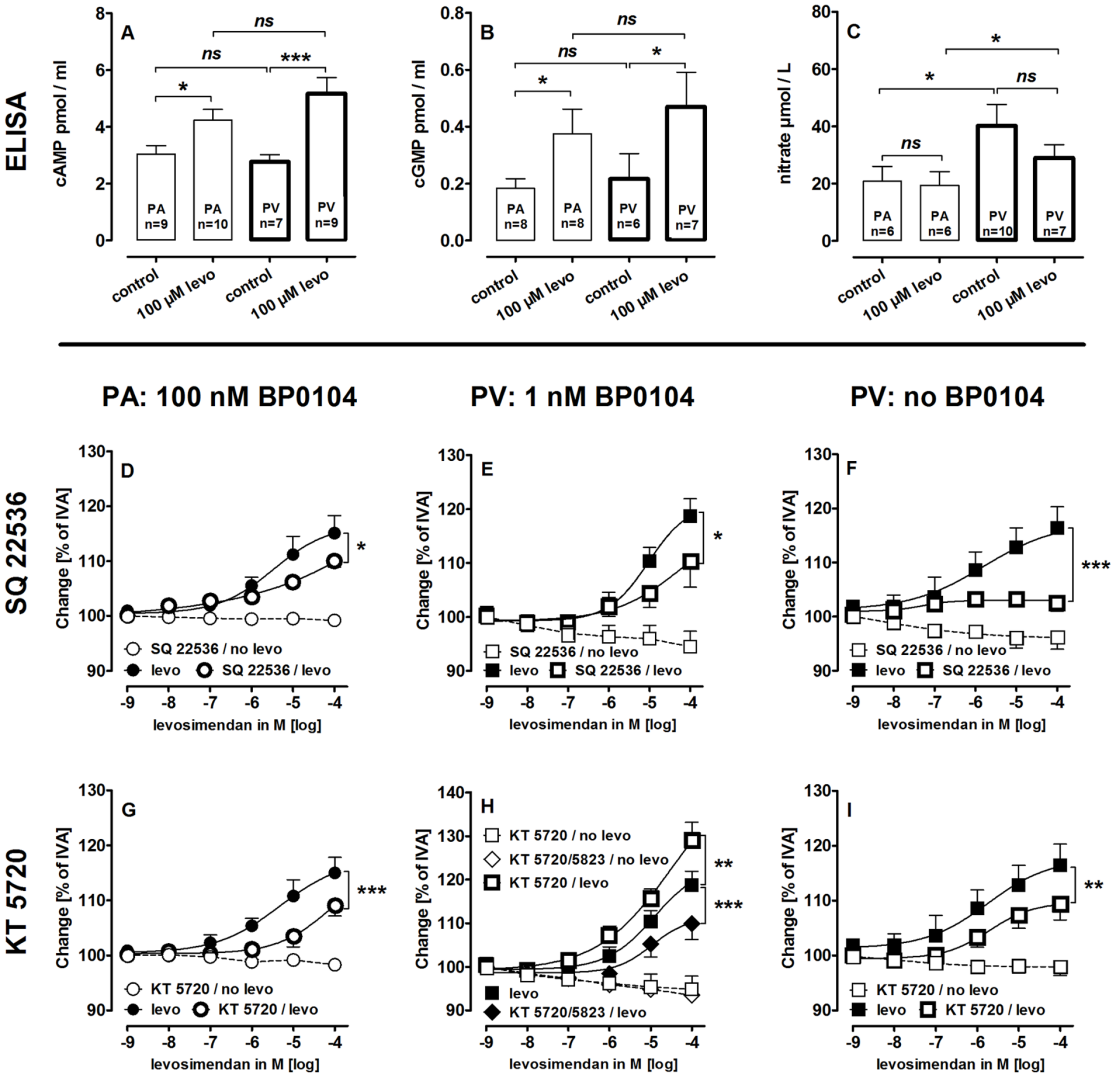
Influence of levosimendan (levo) on cAMP/cGMP and NO-signaling in PAs and PVs. **A)** Effect of levo on cAMP. **B)** Effect of levo on cGMP. **C)** Effect of levo on NO. **D)** **PA (100 nM BP0104):** (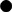) levo (n = 9); ((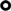) SQ 22536 (100 µM), levo (n = 6); (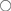) SQ 22526 (100 µM) (n = 4); **E)** **PV (1 nM BP0104):** (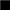) levo (n = 7); (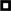) SQ 22536 (100 µM), levo (n = 4); (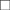 SQ 22536 (100 µM) (n = 3); **F)** **PV:** (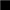) levo (n = 11); (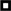) SQ 22536 (100 µM), levo (n = 5); (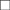) SQ 22536 (100 µM) (n = 4); **G)** **PA (100 nM BP0104):** (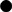) levo (n = 9); (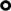) KT 5720 (1 µM), levo (n = 7); (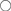) KT 5720 (1 µM) (n = 6); **H)** **PV (1 nM BP0104):** (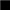) levo (n = 7); (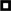) KT 5720 (1 µM), levo (n = 5); (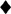) KT 5720 (1 µM), KT 5823 (2 µM), levo (n = 4); (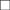) KT 5720 (1 µM) (n = 3); (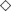) KT 5720 (1 µM), KT 5823 (2 µM) (n = 3); **I)** **PV:** (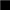) levo (n = 11); (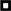) KT 5720 (1 µM), levo (n = 5); (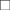) KT 5720 (1 µM) (n = 6); **A–C)** Statistics was performed by the Mann-Whitney U test. **D–I)** Asterics indicate different EC_50_ values. P<0.05 are considered as significant: *p<0.05, **p<0.01 and ***p<0.001.

In isolated PAs and PVs, levosimendan increased intracellular cAMP/cGMP (Fig. 5A/B), whereas NO was not elevated (Fig. 5C). Remarkably, basal NO-levels were higher in the veins.

In PCLS, the role of cAMP was addressed by using the adenyl cyclase-inhibitor SQ 22536 (100 µM) and the protein kinase A (PKA)-inhibitor KT 5720 (1 µM). **Pre-constricted PAs**: SQ 22536 and KT 5720 did not affect BP0104-induced contraction (Table 2), but attenuated levosimendan-induced relaxation (Fig. 5D/G). **Pre-constricted** **and non-pre-constricted PVs**: In both, SQ 22536 and KT 5720 altered neither the BP0104-induced contraction nor the basal tone (Table 2). SQ 22536 reduced the levosimendan-induced relaxation in pre-constricted and non-pre-constricted PVs (Fig. 5E/F). In contrast, KT 5720 only attenuated levosimendan-induced relaxation in control PVs (Fig. 5I), whereas pre-constricted PVs relaxed even stronger (Fig. 5H). Additional treatment with the protein kinase G (PKG)-inhibitor KT 5823 (2 µM) decreased this effect (Fig. 5H).

**Figure 6:**
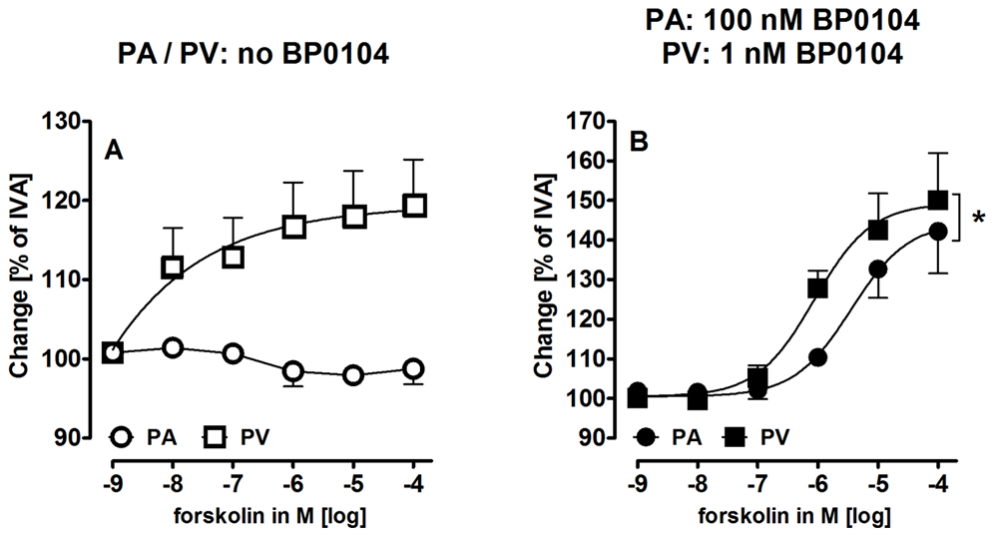
Effect of the adenyl cyclase activator forskolin on PAs and PVs. **A)** Forskolin in control PAs/PVs: (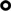) PA (n = 3); (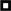) PV (n = 5); **B)** Forskolin in pre-constricted PAs/PVs: (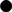) PA (n = 5); (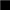) PV (n = 5). Asterics indicate different EC_50_ values. P<0.05 are considered as significant: *p<0.05, **p<0.01 and ***p<0.001.

The significance of cAMP/PKA-signaling for pulmonary vascular relaxation and the potency of adenyl cyclase to relax pulmonary vessels were further studied by the adenyl cyclase activator forskolin. Forskolin relaxed control PVs (Fig. 6A) and pre-constricted PAs/PVs (Fig. 6B), whereas control PAs did not react (Fig. 6A). Of note, pre-constricted PVs relaxed with greater sensitivity to forskolin than pre-constricted PAs with EC_50_-values of 0.86 µM and 3.5 µM respectively.

**Figure 7:**
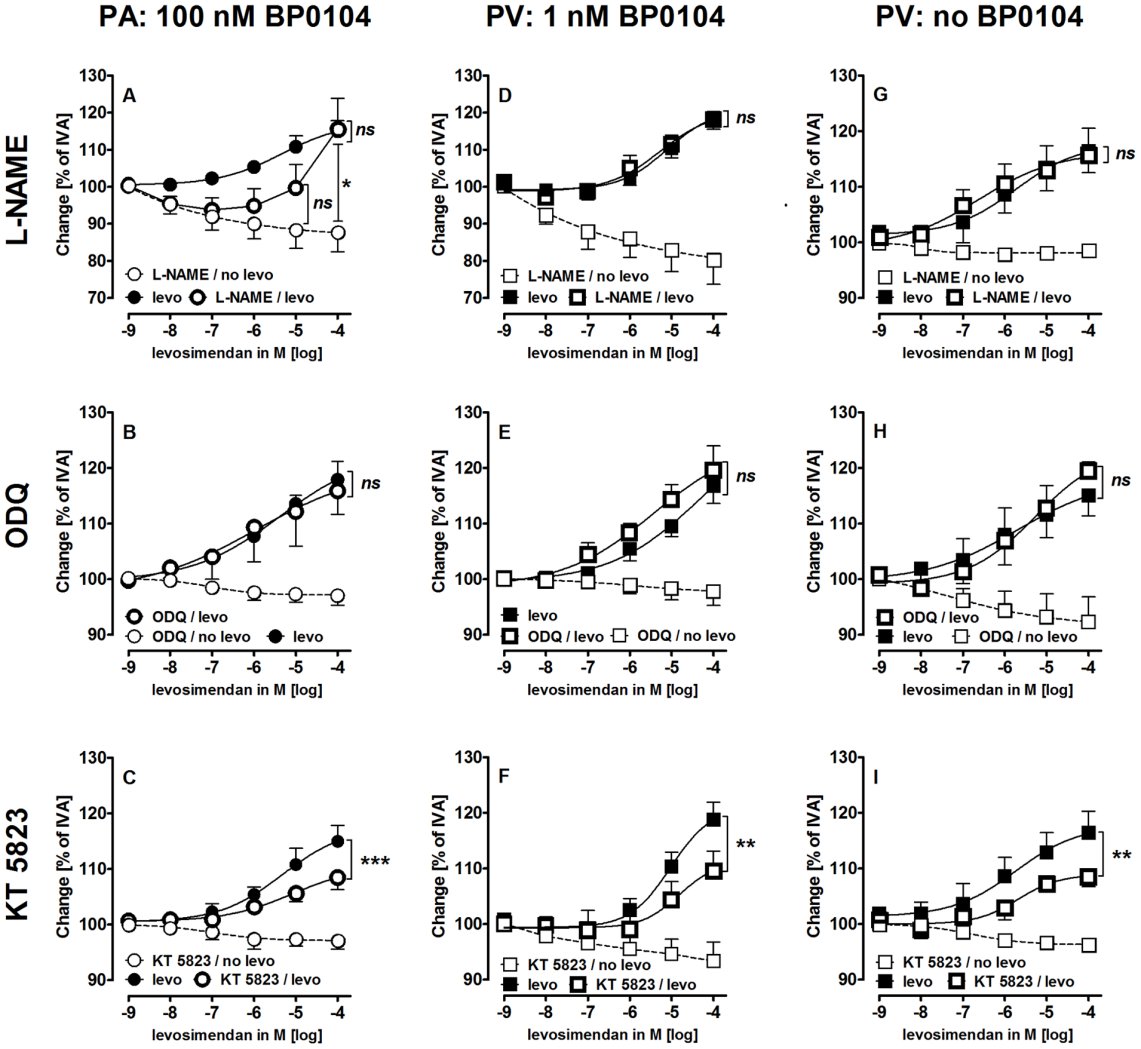
Influence of inhibition of NO/cGMP/PKG-signaling on the relaxant effect of levosimendan (levo). A) PA (100 nM BP0104): (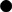) levo (n = 9); (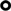) L-NAME (100 µM), levo (n = 9); (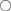) L-NAME (100 µM) (n = 7); B) PA (100 nM BP0104): (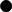) levo (n = 5); (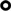) ODQ (1 µM), levo (n = 5); (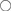) ODQ (1 µM) (n = 3); C) PA (100 nM BP0104): (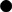) levo (n = 9); (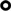) KT 5823 (2 µM), levo (n = 7); (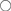) KT 5823 (2 µM) (n = 6); D) PV (1 nM BP0104): (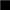) levo (n = 7); (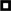) L-NAME (100 µM), levo (n = 6); (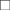) L-NAME (100 µM) (n = 6); E) PV (1 nM BP0104): (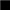) levo (n = 5); (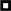) ODQ (1 µM), levo (n = 5); (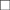) ODQ (1 µM) (n = 4); F) PV (1 nM BP0104): (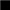) levo (n = 7); (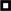) KT 5823 (2 µM), levo (n = 5); (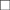) KT 5823 (2 µM) (n = 3); G) PV: (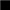) levo (n = 11); (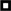) L-NAME (100 µM), levo (n = 8); (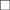) L-NAME (100 µM) (n = 5); H) PV: (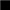) levo (n = 5); (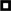) ODQ (1 µM), levo (n = 5); (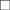) ODQ (1 µM) (n = 4); I) PV: (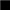) levo (n = 11); (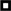) KT 5823 (2 µM), levo (n = 7); (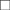) KT 5823 (2 µM) (n = 6); A) Corresponding concentrations were compared by the Mann-Whitney U test. B–I) Asterics indicate different EC_50_ values. P<0.05 are considered as significant: *p<0.05, **p<0.01 and ***p<0.001.

To examine the role of NO, we utilized the NO-synthase-inhibitor L-NAME (100 µM), the guanylyl cyclase-inhibitor ODQ (1 µM) and KT 5823 (2 µM). **PA**: L-NAME did not affect control PAs, but enhanced the contractile effect of BP0104, whereas ODQ and KT 5823 were without effect (Table 2). In pre-constricted PAs, L-NAME provoked a steady contraction that was partially inhibited by levosimendan (Fig. 7A). ODQ did not influence the relaxant effect of levosimendan (Fig. 7B), whereas, KT 5823 reduced it (Fig 7C). **PV**: L-NAME, ODQ and KT 5823 increased the vascular tone (Table 2), but did not affect the BP0104-induced contraction (Table 2). Further, L-NAME and ODQ did not alter the relaxant effect of levosimendan independent from the presence (Fig. 7D/E) or the absence of BP0104 (Fig. 7G/H). In contrast, KT 5823 reduced the relaxant effect of levosimendan in pre-constricted and non-pre-constricted PVs (Fig. 7F/I).

### Interaction of K^+^-channels and cAMP/cGMP on the Tone of PAs and PVs

Apparently, K^+^-channels and cAMP/cGMP contribute to the levosimendan-induced relaxation. To study a possible interaction between K^+^-channels and cAMP/cGMP, pre-constricted vessels were relaxed with the K_ATP_-channel activator levcromakalim (levcrom) and with the BK_Ca_^2+^-channel activator BMS. BMS only slightly relaxed PAs (108%) and had no effect in PVs (not shown). In contrast, levcromakalim relaxed PAs and PVs comparable to 124% and 122% of IVA, respectively. The following experiments with levcromakalim were only done in PAs, as levcromakalim caused an unspecific relaxation in PVs that was not blocked by glibenclamide (data not shown). In addition, the relaxant effects of BMS were too weak for further investigation.

**Figure 8:**
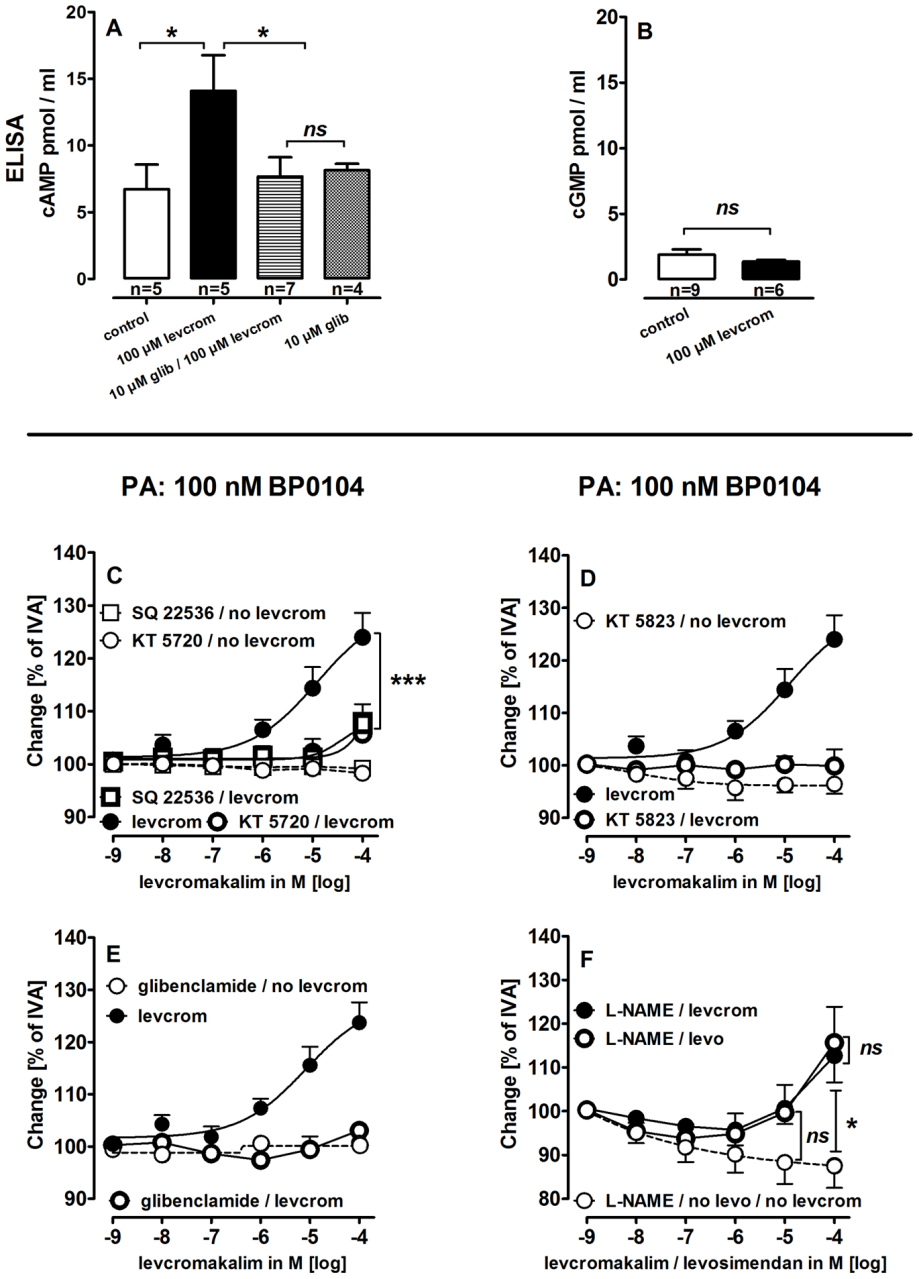
Impact of cAMP/PKA/PKG on K+-channels. **A)** Effect of levcromakalim (levcrom) and glibenclamide on cAMP. **B)** Effect of levcrom on cGMP. **C) PA (100 nM BP0104):** (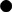) levcrom (n = 6); (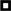) SQ 22536, levcrom (n = 6); (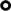) KT 5720, levcrom (n = 6); (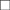) SQ 22536 (100 µM) (n = 4); (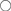) KT 5720 (1 µM) (n = 6); **D)** **PA (100 nM BP0104):** (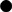) levcrom (n = 6); (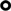) KT 5823 (2 µM), levcrom (n = 4); (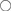) KT 5823 (1 µM) (n = 4); **E) PA (100 nM BP0104):** (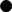) levcrom (n = 7); (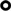) glibenclamide (10 µM), levcrom (n = 3); (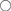) glibenclamide (10 µM) (n = 3); **F)** **PA (100 nM BP0104):** (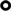) L-NAME (100 µM), levo (n = 9); (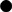) L-NAME (100 µM), levcrom (n = 7); (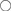) L-NAME (100 µM) (n = 7); **A/B)** Statistics was performed by the Mann-Whitney U test. **C)** Asterics indicate different EC_50_ values. **F)** Corresponding concentrations were compared by the Mann-Whitney U test. P<0.05 are considered as significant: *p<0.05, **p<0.01 and ***p<0.001.

**In PAs**, levcromakalim (100 µM) increased cAMP-levels dependent on K_ATP_-channel activation, (Fig. 8A), whereas cGMP-levels remained unchanged (Fig. 8B). The levcromakalim-induced relaxation was strongly attenuated by inhibition of adenyl cyclase (SQ 22536), PKA (KT 5720) (Fig. 8C), PKG (KT 5823) (Fig. 8D) and K_ATP_-channel-inhibition (glibenclamide) (Fig. 8E). Further, levcromakalim relaxed PAs pre-treated with BP0104 and L-NAME comparable to levosimendan (Fig. 8F).

## Discussion

Levosimendan is used to treat acute heart failure and secondary PH, clinical conditions where relaxation of pulmonary vessels is considered beneficial. Here we demonstrate that levosimendan relaxes PAs and PVs primarily via activation of K_ATP_-channels and the elevation of cAMP and cGMP. In addition, BK_Ca_^2+^- and K_v_-channels appear to be involved in PAs, but not in PVs, demonstrating that the particular mechanisms by which levosimendan acts differ between PAs and PVs.

### The Model

The relaxant effects of levosimendan were studied in the *in vitro* model of PCLS, which is increasingly being used to investigate pulmonary vascular pharmacology.

PCLS offer several possibilities: **1)** PCLS allow studying exclusively PAs and PVs, independent of ventricular contractility and volume load. **2)** PCLS enable to study PAs and PVs at the same time in the same slice. **3)** Contractions in PCLS are auxotonic as *in vivo*, thus the model of PCLS represents a valuable extension to isotonic or isometric studies that are mostly done in isolated vessels. **4)** PCLS can be prepared from various species, including humans and allow thereby an interspecies comparison.

Here, the levosimendan-induced relaxation was studied in normal, but not in diseased pulmonary vessels. However, because ET_A_-receptors are up-regulated in PH [22], we tried to imitate this condition by pre-constriction with BP0104 and in some experiments also by blockade of NO, another characteristic of PH [23]. The differential arterial and venous responses to BP0104 confirm the heterogeneity of the pulmonary vasculature and are in line with findings in porcine vessels [24] and are consistent with own unpublished data in human pulmonary vessels.

### Role of K^+^-channels in Levosimendan-induced Vasorelaxation

**Figure 9:**
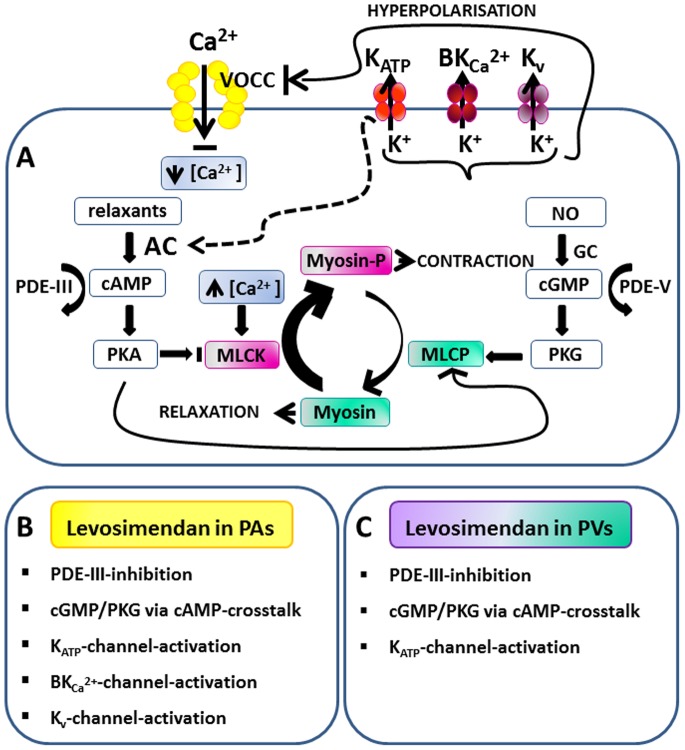
Regulation of vascular smooth muscle cells and the involvement of levosimendan. **A)** Myosin light chain kinase (MLCK) and myosin light chain phosphatase (MLCP) regulate vascular smooth muscle cells (VSMCs). High cytosolic Ca^2+^-levels activate MLCK which phosphorylates myosin light chains (Myosin-p) and thereby enhances VSMC contraction. In contrast, MLCP dephosphorylates Myosin-p and promotes relaxation. MLCP is highly activated by the protein kinase G (PKG) and protein kinase A (PKA). PKA and PKG stimulate K^+^-channels, leading to membrane hyperpolarisation, reduced Ca^2+^-influx via voltage-operated Ca^2+^-channels (VOCC) and reduced cytosolic Ca^2+^. Activation of K_ATP_-channels leads to the production of cAMP, probably by the stimulation of adenyl cyclase (AC). This illustration is modified from Yokoshiki et al. [13]. **B)** Signaling pathways which interact with levosimendan in pulmonary arterial smooth muscle cells (GP). **C)** Signaling pathways which interact with levosimendan in pulmonary venous smooth muscle cells (GP).

In vascular smooth muscle cells (VSMCs), K^+^-channels can become activated either directly, e.g. by levcromakalim, or an interaction of various stimuli, including ROS, hypoxia, Ca^2+^, cAMP/PKA, NO/cGMP/PKG and ATP. Activation of K^+^-channels hyperpolarises the cell membrane and inhibits the cytosolic Ca^2+^-influx via voltage-operated Ca^2+^-channels (VOCC) [25]. Low cytosolic Ca^2+^ levels prevent the activation of myosin light chain kinase (MLCK) [26] and promote relaxation (Fig. 9A).

Here we have studied the three major types of K^+^-channels, namely K_ATP_-, BK_Ca_^2+^- and K_v_-channels. Among them, K_ATP_-channels play the most important role in mediating the pulmonary vascular effects of levosimendan (Fig. 9B/C), whereas BK_Ca_^2+^- and K_v_-channels did contribute only in pre-constricted PAs (Fig. 9B). This fact is notably, as K_ATP_-channel-inhibition (glibenclamide) alone already completely prevented levosimendan-induced relaxation and allows two assumptions 1) glibenclamide blocks aside K_ATP_-channels also BK_Ca_^2+^- and K_v_-channels; 2) the role of K_ATP_-channels is dominant and the impact of BK_Ca_^2+^- and K_v_-channels less, that the soley inhibition of K_ATP_-channels might be sufficient to prevent the relaxant properties of levosimendan. With regard to assumption 1) it might be refused, as glibenclamide does not inhibit BK_Ca_^2+^-or K_v_-channels [26]. Concerning assumption 2) the inferior role of BK_Ca_^2+^-channels in levosimendan-induced relaxation is supported by the observation that activation of BK_Ca_^2+^-channels by the BK_Ca_^2+^-channel opener BMS only slightly relaxed PAs. In contrast, the K_ATP_-channel opener levcromakalim exerted a pronounced relaxant effect in PAs (124%). However, from our experiments; we still would expect a slight relaxant effect of levosimendan in PAs despite K_ATP_-channel inhibition. Finally, we cannot solve the precise contribution of BK_Ca_^2+^-channels to levosimendan-induced relaxation. In principle, levosimendan-induced activation of BK_Ca_^2+^-channels has been reported for porcine coronary arteries [9] and for human thoracic arteries [10]. Hence, it is also conceivable for large PAs, which are well-known densely equipped with BK_Ca_^2+^-channels [27]. With regard to the contribution of K_v_-channels to levosimendan-induced relaxation, we did not study the impact of K_v_-channel activation in pulmonary vascular relaxation, as no suitable activators are available.

In PVs, K_v_-channel-inhibition did not influence the relaxant effect of levosimendan, but raised the tone of control PVs illustrating their role in the regulation of the pulmonary venous tone [28]. The missing effects of 4-aminopyridine on the BP0104-induced contraction might be due to the activation of protein kinase C, which in turn inhibits K_v_-channels [29]. Clearly, PAs and PVs differ in the regulation of their tone and in the role of the individual K^+^-channels in response to levosimendan.

### Relevance of cAMP/PKA-signaling on Levosimendan-induced Vasorelaxation

Cyclic AMP – via activation of PKA – can relax smooth muscle by increasing myosin light chain phosphatase (MLCP)-activity [30], by blocking MLCK [31] and by stimulating K^+^-channels [26] (Fig. 9A). In line with data from coronary arteries [32], we have found that levosimendan increases cAMP in PAs/PVs. The functional relevance of this cAMP-increase was shown by the observation that inhibition of adenyl cyclase or PKA reduced the levosimendan-induced relaxation in PAs/PVs as well as by the relaxant effects of forskolin. Somewhat unexpected, PKA-inhibition had no effect in PVs though, a finding that might be explained by the role of other relaxant mediators such as cGMP/PKG or by the existence of side-effects from the activation of ET_A_-receptors by BP0104 such as an excess of PKA [33], [34] or of PKG [35]. It seems also possible that PKA and PKG interact in a non-linear fashion, which would be consistent with the observation that simultaneous inhibition of PKA and PKG did largely attenuate the levosimendan-induced relaxation. Taken together, our findings demonstrate that the cAMP-PKA axis contributes to the levosimendan-induced relaxation and suggest that levosimendan elevates cAMP either by inhibiting relevant PDE-isoenzymes at ≤1 µM or by an unknown mechanism that is dependent on K_ATP_-channels (see below).

### NO/cGMP/PKG-signaling in Levosimendan-induced Vasorelaxation

The NO/cGMP/PKG-pathway plays a dominant role in VSMC relaxation (Fig. 9A). PKG promotes Ca^2+^-desensitation via MLCP-activation [31] and stimulates K^+^-channels.

Levosimendan failed to increase NO-levels in pulmonary vessels (Fig. 5C); and in line L-NAME and ODQ also failed to attenuate the levosimendan-induced vasodilation (Fig. 7). Interestingly, levosimendan increased cGMP in PVs and PAs despite any effect on NO-synthesis. Further inhibition of PKG by KT 5823 attenuated the relaxant effect of levosimendan. Hence the origin of cGMP might be explained by a cross-talk between the cAMP/PKA- and the NO/cGMP/PKG-pathway in VSMCs which exists on various levels [31], [36], [37]. Our data are supported by studies in coronary vessels that were relaxed by levosimendan and that showed slightly increased cGMP-levels [38] despite the lack of endothelium and thus eNOS-synthase [32]. The functional role of cGMP in pulmonary vessels was clearly shown by the finding that PKG-inhibition attenuated the levosimendan-induced vasodilation in PAs and PVs (Fig. 7C/F/I).

### The Relative Importance of K^+^-channels, cAMP/PKA/PKG for Levosimendan-induced Relaxation

We have demonstrated that the levosimendan-induced relaxation of pulmonary vessels is mainly based on K_ATP_-channel-activation and cAMP/cGMP-production. These findings raise the question whether K_ATP_-channels and cAMP/cGMP act additive or in a sequential manner. We therefore studied the effects of the K_ATP_-channel opener levcromakalim on vessel tone and cyclic nucleotides (Fig. 8). Surprisingly, levcromakalim increased cAMP and that response was ablated by glibenclamide, indicating that activation of K_ATP_-channels may somehow stimulate cAMP-production. This is a novel observation that requires further study. The relevance of this phenomenon is demonstrated by the finding that inhibition of adenyl cyclase and PKA blocked the relaxant effect of levcromakalim showing that the activation of K_ATP_-channels relaxes PAs via cAMP and PKA. Notably, inhibition of PKG also blocked the relaxant effect of levcromakalim, although levcromakalim did not affect cGMP-levels, an observation which might be explained by cAMP-dependent activation of PKG [39].

### Differential behaviour of PAs and PVs

Recently we showed that PAs and PVs respond differently to β-receptor stimulation [16]. Here we report further differences between PAs and PVs e.g. control PVs relaxed to levosimendan, isoproterenol or forskolin, whereas control PAs did not. Further, levosimendan increased cAMP stronger in PVs (87%) than in PAs (39%). Since basal cAMP-levels were comparable, this may indicate that PVs contain more PDE (probably PDE-III) than PAs. Further, the increase of cAMP/cGMP in control PAs may at least in part explain why levosimendan prevents the contractile effect of epinephrine in PAs. In addition to the differences in cAMP/PKA-signaling, NO-levels were higher in PVs than in PAs (Fig. 5) and in line with this, NOS inhibition did contract PVs only (Table 2). These findings suggest the particular importance of NO-signaling in PVs corroborating previous findings in human PVs [40] and porcine pulmonary vessels [41].

The differential relaxant behaviour of control PVs and PAs proposes that control PVs exhibit a certain resting tone. From our results with fasudil we conclude that Ca^2+^-sensitization contributes to maintain this resting tone, whereas NO and K_v_-channels may counteract this response. Obviously, cAMP-providing agents such as levosimendan, isoproterenol or forskolin, but also NO [16] might overcome this resting tone and relax PVs. Based on these data and considerations, levosimendan appears to oppose the resting tone by cAMP/cGMP, which finally all counteract Ca^2+^-sensitization [30], [31].

In view of a certain resting tone, we need to consider mechanical forces which may generate such a resting tone. In PCLS, the tone of PAs, PVs and airways is influenced by the surrounding tissue, whereas the load of the surrounding tissue is primarily determined by the agarose filled in the lung [42]. PAs, PVs and airways lie always in the center of the slices, therefore the load of the embedding tissue should affect PAs, PVs and airways similarly and the tethering forces should be comparable. Therefore it appears unlikely that a certain resting tone of PVs depends on the slice preparation.

This study in guinea pigs demonstrates that levosimendan relaxes large PAs and PVs up to 119%. Prima facie, the relaxant effect of levosimendan appears to be marginal. However taken into consideration the Hagen-Poiseuille law, the flow resistance increases 16 fold, if the radius divides in half. Finally, the presented vascular effects of levosimendan are sufficient pronounced to be relevant for pulmonary vascular resistance. These experiments were performed in central pulmonary vessels which primarily do not contribute to pulmonary vascular resistance; from own preliminary human data we know that levosimendan also relaxes small human pulmonary vessels potently which definitely contribute to pulmonary vascular resistance. In this study, the required concentrations for levosimendan-induced relaxation were 1 µM in pre-constricted PAs/PVs and 320 nM in non-pre-constricted PVs. In patients, plasma concentrations of 850 nM levosimendan are reached [43], indicating that the present findings may be clinically relevant.

In conclusion, this study shows that levosimendan relaxes PAs and PVs by different mechanisms. Clinically, this suggests the use of levosimendan in the therapy of increased right ventricular afterload due to right heart failure. In left heart failure, the pulmonary venous relaxant effects of levosimendan might act synergistic to its well-known positive inotropic effects, as reduced hydrostatic pressures alleviate lung edema, left ventricular volume overload and secondary PH. If the pulmonary relaxant effects of levosimendan could be proven in PA and PVs from a PH-disease model, levosimendan might become of potential interest in the therapy of PH and pulmonary veno-occlusive disease.
